# To the Editors of the Medical and Physical Journal

**Published:** 1806-11

**Authors:** Thomas Key

**Affiliations:** Fenchurch Street


### *To the Editors of the Medical and Physical Journal.*

GENTLEMEN,

WILL you do me the favour of inserting in the next Number of the Medical and Physical Journal, the following Reply to an assertion of Mr. Ring's, published in No. xcii. p. 325, of that useful publication.

Yours, respectfully,

*Fenchurch Street, Oct.* 20, 1806. THOMAS KEY.

---

Mr. RING, in his Remarks upon some cases of supposed Cow-pock failure, which I some time since transmitted him, appears to have mistaken the motive that induced me to make the communication, by which he has implicated me as the enemy of Vaccination. What must the parents of those children whom I have vaccinated infer, when, after having resisted every intreaty for variolous inoculation, they witness my name among the opponents of cow-pock, and find me engaged in substantiating evidence that it is only a temporary protection against small-pox? My practice stands in high opposition to any evidence of the kind: and the influence I possess has, from a conviction of its security, been diligently employed in sounding the praise of that inestimable discovery. Mr. Ring, by referring to the documents he speaks of, will, I am confident, do me the justice to say, that there is nothing to sanction the assertion, "That the intention of the communication was to bring forward evidence in addition to what has been already advanced by some authors, that vaccination is only a temporary protection against the small-pox." That the cases to which those documents refer did not occur in my own practice, is a fact; but I collected them with care, and can speak to their veracity; they appeared to contain occurrences deserving of consideration, and under that impression only I made the communication.

---

